# Preoperative OCT biomarkers and surgical outcomes after vitrectomy with epiretinal membrane and internal limiting membrane peeling for refractory diabetic macular edema: a retrospective cohort study

**DOI:** 10.1186/s12886-026-05352-0

**Published:** 2026-09-22

**Authors:** Ozge Saritas, Elif Sahin, Yelda Yildiz Tasci, Yasin Toklu

**Affiliations:** 1 Department of Ophthalmology, Ankara Yildirim Beyazit University Faculty of Medicine, Ankara, Türkiye; 2 Department of Ophthalmology, Ankara Bilkent City Hospital, Ankara, Türkiye

**Keywords:** Diabetic macular edema, Pars plana vitrectomy, Epiretinal membrane, Internal limiting membrane, Optical coherence tomography, Refractory diabetic macular edema

## Abstract

**Background:**

Vitrectomy with epiretinal membrane (ERM) and internal limiting membrane (ILM) peeling may provide anatomical and functional benefit in selected eyes with refractory diabetic macular edema, but predictors of postoperative outcome remain incompletely defined. This study aimed to evaluate the anatomical and functional outcomes of pars plana vitrectomy (PPV) combined with ERM and ILM peeling in eyes with diabetic macular edema (DME) and to investigate the prognostic value of preoperative OCT biomarkers for postoperative outcomes.

**Methods:**

Thirty eyes of 30 patients with DME refractory to repeated intravitreal anti-VEGF and/or corticosteroid therapy underwent 25-gauge PPV with ERM and ILM peeling. Best-corrected visual acuity (BCVA), central subfield thickness (CST), postoperative treatment requirement, and preoperative OCT biomarkers were evaluated. Associations between baseline OCT biomarkers and 12-month anatomical and functional outcomes were assessed using unadjusted analyses and multivariable regression models.

**Results:**

Mean BCVA improved from 1.08 ± 0.52 logMAR at baseline to 0.65 ± 0.42 logMAR at 12 months (*P* < 0.001), while mean CST decreased from 533.80 ± 100.31 μm to 261.23 ± 34.31 μm (*P* < 0.001). Baseline DRIL was independently associated with worse 12-month BCVA (β = 0.399, 95% CI 0.035–0.762; *P* = 0.032). Eyes with intact EZ showed greater numerical visual improvement than those with EZ disruption, although the difference was not statistically significant (*P* = 0.093). Absence of baseline HRF was associated with greater CST reduction in the unadjusted analysis (*P* < 0.001), but HRF was not independently associated with 12-month CST (*P* = 0.309). During postoperative follow-up, 22 eyes (73.3%) required no additional intravitreal treatment.

**Conclusions:**

PPV with ERM and ILM peeling was associated with significant anatomical and functional improvement in selected eyes with refractory DME. Among the preoperative OCT biomarkers evaluated, DRIL showed the most consistent prognostic association with postoperative visual outcome. Preoperative assessment of retinal microstructure may provide complementary information for visual prognostication and patient counseling.

## Background

Diabetic macular edema (DME) remains one of the leading causes of vision loss in working-age individuals with diabetes [1, 2]. Although intravitreal pharmacotherapy has substantially improved the management of DME, a considerable proportion of patients continue to experience persistent or recurrent macular edema requiring repeated treatment [3, 4]. In routine clinical practice, some patients show an inadequate response despite multiple intravitreal anti-VEGF and/or corticosteroid injections, often referred to as refractory DME [5, 6]. In these eyes, persistent macular thickening and limited functional improvement despite intensive treatment highlight the need for alternative therapeutic strategies [7].

In selected patients, particularly those with epiretinal membrane (ERM) or vitreomacular interface abnormalities, pars plana vitrectomy (PPV) combined with ERM and internal limiting membrane (ILM) peeling has emerged as a potential therapeutic alternative [8, 9]. Beyond relieving tangential traction, vitrectomy may enhance retinal oxygenation, facilitate the clearance of inflammatory cytokines and vascular endothelial growth factor (VEGF) from the vitreous cavity, improve intraocular drug diffusion, and reduce chronic mechanical stress on the macula [10, 11]. Nevertheless, the reported anatomical and functional outcomes following PPV for refractory DME remain heterogeneous, indicating that careful patient selection remains essential [12]. Therefore, accurate identification of patients most likely to respond to surgery has become increasingly important.

Optical coherence tomography (OCT) has transformed the evaluation of retinal diseases by enabling detailed assessment of retinal microstructure beyond retinal thickness measurements. Recent studies and systematic reviews have identified several baseline OCT biomarkers, including ellipsoid zone (EZ) integrity, external limiting membrane (ELM) integrity, disorganization of the retinal inner layers (DRIL), and hyperreflective retinal foci (HRF), as important predictors of visual outcomes and treatment response in eyes with diabetic macular edema [13–17]. However, evidence regarding their prognostic significance in patients undergoing vitrectomy for refractory DME remains limited [18]. Whether these preoperative OCT biomarkers can predict postoperative anatomical recovery, visual improvement, and subsequent treatment burden after surgery has not been fully elucidated.

Therefore, the present study aimed to evaluate the anatomical and functional outcomes of PPV combined with ERM and ILM peeling in eyes with refractory DME and to investigate the prognostic value of preoperative OCT biomarkers for postoperative visual and anatomical outcomes. We further sought to determine whether these biomarkers could provide additional information for preoperative visual prognostication and patient counseling.

## Methods

### Study design and patients

This retrospective consecutive cohort study included 30 eyes of 30 patients with refractory diabetic macular edema (DME) who underwent pars plana vitrectomy (PPV) with epiretinal membrane (ERM) and internal limiting membrane (ILM) peeling at a tertiary referral center. Only eyes with OCT-confirmed ERM and a minimum postoperative follow-up of 12 months were included. ERM was defined on spectral-domain optical coherence tomography (SD-OCT) as a hyperreflective fibrocellular membrane along the inner retinal surface associated with retinal surface wrinkling, distortion of the foveal contour, or retinal thickening. Baseline ERM severity was retrospectively graded on preoperative SD-OCT images according to the OCT-based staging system described by Govetto et al. [19]. In this system, stage 3 ERM is characterized by continuous ectopic inner foveal layers with otherwise identifiable retinal layers, whereas stage 4 additionally demonstrates marked disorganization of the retinal layers. ERM staging was performed independently by two experienced retina specialists who were masked to postoperative outcomes, with disagreements resolved by consensus.

The study adhered to the principles of the Declaration of Helsinki and complied with the Strengthening the Reporting of Observational Studies in Epidemiology (STROBE) recommendations. Approval was obtained from the Ankara Bilkent City Hospital Ethics Committee (Approval No. TABED 2-26-1899). Owing to the retrospective nature of the study, the requirement for informed consent was waived by the Ethics Committee.

Refractory DME was defined as persistent center-involving DME despite at least three consecutive intravitreal anti-vascular endothelial growth factor (anti-VEGF) injections, with or without corticosteroid therapy, accompanied by persistent intraretinal and/or subretinal fluid and inadequate anatomical or functional improvement. Preoperative intravitreal treatment history was reviewed for each eye, including the anti-VEGF agent or agents administered, the number of anti-VEGF injections, previous intravitreal dexamethasone implant treatment, and the interval between the last intravitreal treatment and surgery. Anti-VEGF injections and dexamethasone implants were recorded separately, and cumulative preoperative intravitreal treatment burden included both treatment types.

Baseline diabetic retinopathy severity was classified as non-proliferative diabetic retinopathy (NPDR) or proliferative diabetic retinopathy (PDR). The presence of tractional retinal detachment (TRD) and fibrovascular proliferation (FVP) was specifically assessed from the preoperative clinical examination and retinal imaging.

Exclusion criteria included vitreomacular traction (VMT), full-thickness macular hole, previous vitreoretinal surgery, retinal vascular or inflammatory diseases other than diabetic retinopathy, inherited retinal disorders, significant media opacity precluding adequate OCT imaging, and incomplete follow-up. VMT was defined as persistent focal or broad vitreous attachment to the macula associated with anteroposterior traction causing distortion of the foveal surface and/or intraretinal structural changes. Tangential retinal distortion attributable to ERM in the absence of persistent vitreous attachment was not classified as VMT.

### Surgical technique

All procedures were performed by the same experienced vitreoretinal surgeon using a standardized surgical technique. A standard 25-gauge three-port transconjunctival PPV was performed under local anesthesia using the Constellation Vision System (Alcon Laboratories, Fort Worth, TX, USA). Following core vitrectomy, posterior vitreous detachment was induced when not already present, and meticulous peripheral vitrectomy was completed. ERM peeling was performed using end-gripping forceps, followed by ILM peeling in all eyes. Brilliant Blue G dye was used to facilitate visualization of the ILM, which was peeled in a circular fashion centered on the fovea and extending approximately 2–3 disc diameters. No eye underwent ERM peeling with preservation of the ILM.

Combined phacoemulsification with intraocular lens implantation was performed in phakic eyes when clinically indicated. At the conclusion of surgery, a thorough peripheral retinal examination was performed to identify any iatrogenic retinal breaks. Fluid–air exchange was routinely performed at the conclusion of surgery as part of the surgeon’s standardized surgical protocol, and the vitreous cavity was subsequently left filled with air. Postoperative treatment consisted of standard topical antibiotic and corticosteroid therapy.

### Ophthalmic examination and OCT analysis

All patients underwent comprehensive ophthalmic examination at baseline and during postoperative follow-up. Best-corrected visual acuity (BCVA) was measured using Snellen charts and converted to logarithm of the minimum angle of resolution (logMAR) units for statistical analysis. Slit-lamp biomicroscopy and dilated fundus examination were performed in all cases. SD-OCT imaging was obtained using the same device for all patients (Heidelberg Engineering, Heidelberg, Germany) under standardized acquisition protocols. Central subfield thickness (CST) was calculated using the built-in software based on the Early Treatment Diabetic Retinopathy Study (ETDRS) grid. Automated retinal segmentation and CST measurements were reviewed for all scans by two experienced retina specialists, and segmentation errors were manually corrected when necessary before analysis.

Preoperative OCT images were evaluated for retinal microstructural biomarkers potentially associated with postoperative outcomes. Ellipsoid zone (EZ) integrity at the foveal center was classified as intact or disrupted. Disorganization of the retinal inner layers (DRIL) was defined as the inability to identify the boundaries between the ganglion cell–inner plexiform layer complex, inner nuclear layer, and outer plexiform layer within the central 1-mm zone. The cystoid edema pattern was categorized as focal or diffuse according to the distribution and extent of intraretinal cystic spaces. Subfoveal hyperreflective retinal foci (HRF) within the central 1-mm zone were recorded as present or absent.

Postoperative OCT examinations were performed at 1, 3, 6, and 12 months. EZ, DRIL, and HRF status were additionally reassessed at 12 months to evaluate longitudinal changes in these biomarkers. All OCT images were independently evaluated by two experienced retina specialists who were masked to postoperative outcomes, and disagreements were resolved by consensus.

### Postoperative follow-up and retreatment

All included eyes completed at least 12 months of postoperative follow-up. The prespecified 12-month examination was used for the principal functional and anatomical outcome analyses, whereas additional follow-up beyond 12 months was used to characterize overall follow-up duration and treatment burden. OCT data were available for all 30 eyes at each prespecified postoperative time point.

Postoperative intravitreal retreatment was considered in eyes with persistent or recurrent center-involving intraretinal and/or subretinal fluid on OCT, particularly when accompanied by deterioration or insufficient improvement in BCVA. Treatment decisions were individualized by the treating retina specialist after exclusion of other potential causes of visual loss. The number of additional intravitreal treatments administered during postoperative follow-up was recorded for each eye.

### Statistical analysis

Statistical analyses were performed using Python version 3.12 (Python Software Foundation, Wilmington, DE, USA). Continuous variables are presented as mean ± standard deviation (SD), and categorical variables as number and percentage. Normality was assessed using the Shapiro–Wilk test. Changes in central subfield thickness (CST) from baseline to postoperative months 1, 3, 6, and 12 were evaluated using paired-samples t-tests. The corresponding change scores did not significantly deviate from normality (Shapiro–Wilk *P* = 0.269, *P* = 0.357, *P* = 0.257, and *P* = 0.229, respectively). Because the change in best-corrected visual acuity (BCVA) from baseline to 12 months was not normally distributed (Shapiro–Wilk *P* < 0.001), this comparison was performed using the Wilcoxon signed-rank test. For unadjusted analyses of baseline OCT biomarkers, visual improvement was defined as baseline minus 12-month logMAR BCVA and was compared according to ellipsoid zone (EZ), disorganization of the retinal inner layers (DRIL), and hyperreflective retinal foci (HRF) status using two-sided Mann–Whitney U tests. Anatomical improvement was defined as baseline minus 12-month CST. CST reduction values within the baseline OCT biomarker subgroups were consistent with normality (all Shapiro–Wilk *P* > 0.23); therefore, between-group anatomical comparisons were performed using two-sided Welch’s t-tests. Changes in the prevalence of EZ disruption, DRIL, and HRF between baseline and 12 months were evaluated using exact McNemar tests. To assess whether baseline OCT biomarkers were independently associated with postoperative outcomes, separate parsimonious multivariable linear regression models were constructed for each biomarker. Models evaluating 12-month BCVA were adjusted for baseline BCVA, concomitant phacoemulsification, and diabetic retinopathy stage, whereas the model evaluating 12-month CST was adjusted for baseline CST, concomitant phacoemulsification, and diabetic retinopathy stage. Heteroscedasticity-consistent HC3 robust standard errors were used. Separate biomarker-specific models were selected to limit overfitting given the sample size. In sensitivity analyses, baseline ERM stage was additionally included in the model evaluating the association between DRIL and 12-month BCVA, and functional biomarker associations were separately assessed among eyes that were pseudophakic at baseline. All statistical tests were two-sided, and *P* < 0.05 was considered statistically significant. Multivariable regression results are reported as regression coefficients (β) with 95% confidence intervals (CIs).

## Results

### Baseline characteristics

A total of 30 eyes of 30 patients with refractory DME were included in the study. All eyes completed at least 12 months of postoperative follow-up. The mean follow-up duration was 14.83 ± 4.22 months, with a minimum follow-up of 12 months. The mean age was 66.43 ± 6.08 years, and 20 patients (66.7%) were male. At baseline, 15 eyes (50.0%) had NPDR and 15 eyes (50.0%) had PDR. No eye had tractional retinal detachment or fibrovascular proliferation. All eyes had advanced ERM, with 15 eyes (50.0%) classified as stage 3 and 15 eyes (50.0%) as stage 4. Eighteen eyes (60.0%) were pseudophakic at baseline, and concomitant phacoemulsification was performed in 10 eyes (33.3%).

All eyes had previously received intravitreal bevacizumab. Four eyes (13.3%) had additionally received aflibercept, and 16 eyes (53.3%) had received intravitreal dexamethasone implants. The mean number of preoperative anti-VEGF injections was 3.97 ± 1.43 (range, 3–6), whereas the mean cumulative number of preoperative intravitreal treatments, including dexamethasone implants, was 5.60 ± 2.36 (range, 1–11). The mean interval between the last intravitreal treatment and surgery was 4.0 months (range, 3–6 months) (Table 1).

**Table 1:** Baseline demographic and clinical characteristics of the patients

Characteristic	Value
Age, years	66.43 ± 6.08
Male sex, n (%)	20 (66.7)
Follow-up duration, months	14.83 ± 4.22
Baseline BCVA, logMAR	1.08 ± 0.52
Baseline CST, µm	533.80 ± 100.31
Phakic eyes at baseline, n (%)	12 (40.0)
Combined phacoemulsification with PPV, n (%)	10 (33.3)
Diabetic retinopathy stage, NPDR/PDR, n (%)	15 (50.0) / 15 (50.0)
ERM stage, stage 3/stage 4, n (%)	15 (50.0) / 15 (50.0)
EZ disruption, n (%)	18 (60.0)
DRIL present, n (%)	22 (73.3)
HRF present, n (%)	22 (73.3)
Cystoid edema pattern, focal/diffuse, n (%)	11 (36.7) / 19 (63.3)
Previous bevacizumab treatment, n (%)	30 (100)
Additional aflibercept treatment, n (%)	4 (13.3)
Previous dexamethasone implant treatment, n (%)	16 (53.3)
Preoperative anti-VEGF injections, number	3.97 ± 1.43
Total preoperative intravitreal treatments, number*	5.60 ± 2.36
Interval from last intravitreal treatment to surgery, months	4.0 (range, 3–6)

Data are presented as mean ± standard deviation or n (%), unless otherwise indicated

*Total preoperative intravitreal treatments include anti-VEGF injections and intravitreal dexamethasone implants

BCVA, best-corrected visual acuity; CST, central subfield thickness; DRIL, disorganization of the retinal inner layers; ERM, epiretinal membrane; EZ, ellipsoid zone; HRF, hyperreflective retinal foci; NPDR, non-proliferative diabetic retinopathy; PDR, proliferative diabetic retinopathy; PPV, pars plana vitrectomy

### Anatomical outcomes

**Fig. 1:**
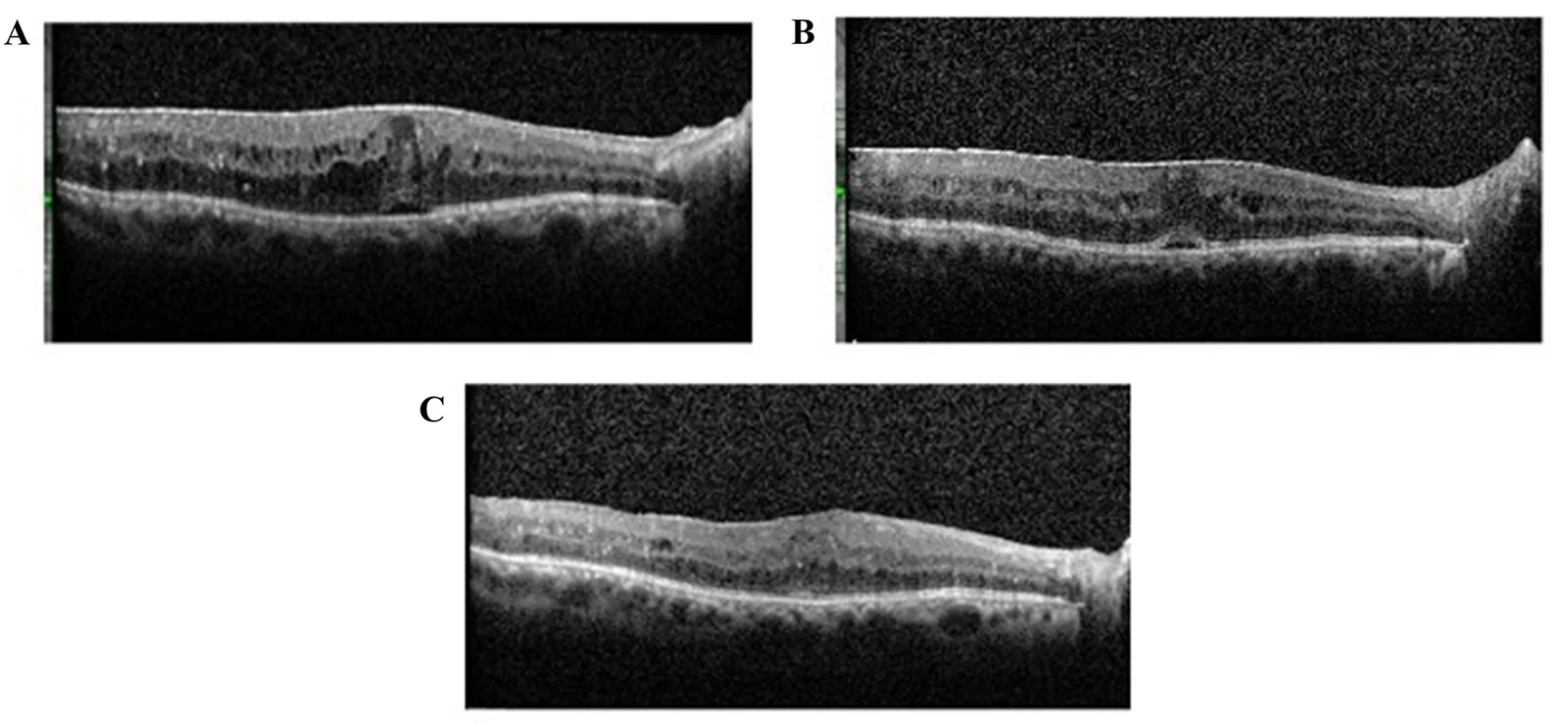
Representative OCT images of an eye with refractory diabetic macular edema. (**A**) Baseline OCT showing persistent macular edema. (**B**) OCT obtained after multiple intravitreal injections demonstrating persistent edema. (**C**) OCT at the final postoperative visit following pars plana vitrectomy with epiretinal membrane and internal limiting membrane peeling. BCVA improved from 1.3 to 0.3 logMAR, and CST decreased from 420 μm to 240 μm

**Fig. 2:**
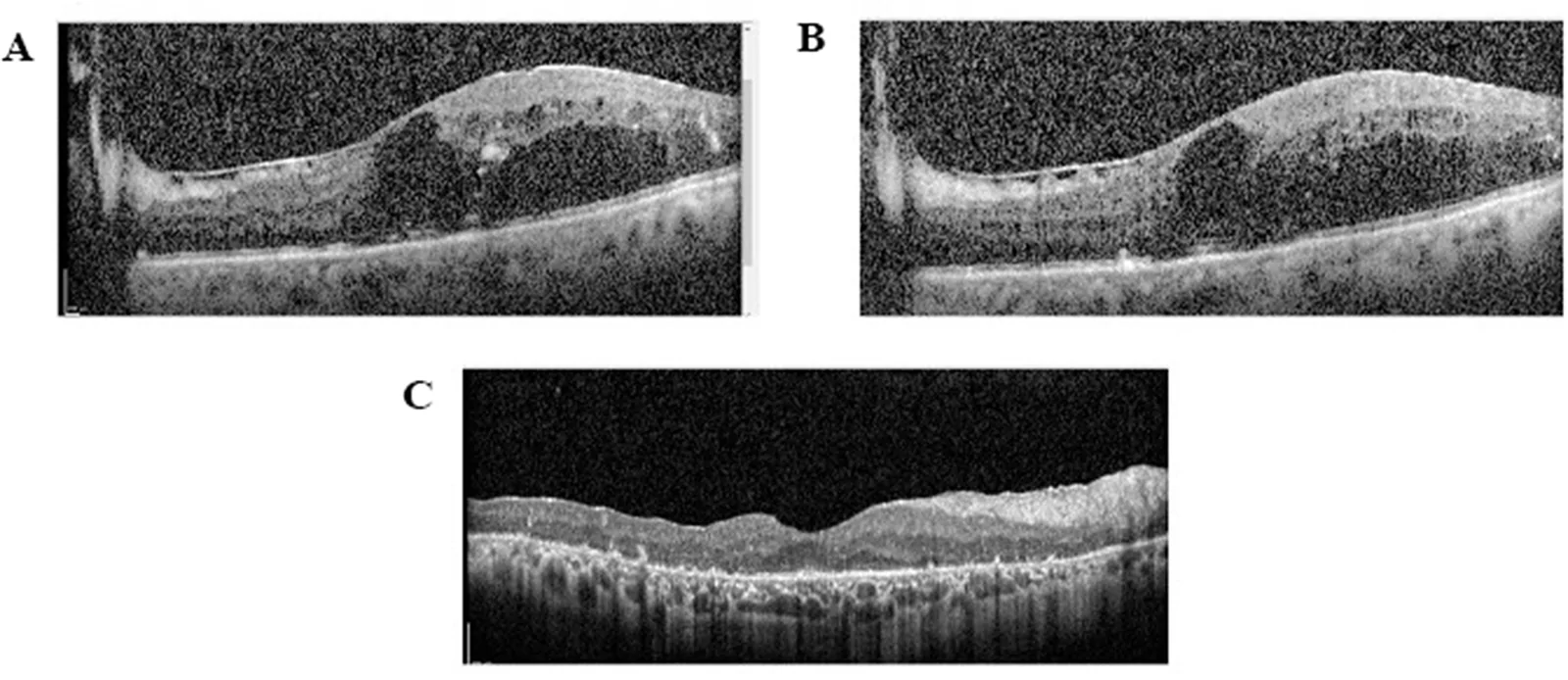
Representative OCT images of a second eye with refractory diabetic macular edema. (**A**) Baseline OCT demonstrating increased central retinal thickness. (**B**) OCT obtained after at least three intravitreal anti-VEGF injections showing persistent edema. (**C**) OCT at the final postoperative visit following pars plana vitrectomy with epiretinal membrane and internal limiting membrane peeling. BCVA improved from 1.3 to 0.8 logMAR, and CST decreased from 557 μm to 248 μm

Mean CST decreased from 533.80 ± 100.31 μm at baseline to 295.63 ± 68.47 μm at 1 month, 293.70 ± 64.00 μm at 3 months, 285.13 ± 54.71 μm at 6 months, and 261.23 ± 34.31 μm at 12 months. CST was significantly lower than baseline at each postoperative time point (all *P* < 0.001) (Table 2). At baseline, 11 eyes (36.7%) exhibited a focal cystoid pattern and 19 eyes (63.3%) a diffuse pattern. Mean BCVA improved from 1.08 ± 0.52 logMAR at baseline to 0.65 ± 0.42 logMAR at 12 months (*P* < 0.001) (Table 3). Representative OCT images illustrating the anatomical and functional course after surgery are shown in Figs. 1 and 2.

**Table 2:** Central subfield thickness (CST) during postoperative follow-up

Time point	CST (µm)	*P* value*
Baseline	533.80 ± 100.31	–
1 month	295.63 ± 68.47	< 0.001
3 months	293.70 ± 64.00	< 0.001
6 months	285.13 ± 54.71	< 0.001
12 months	261.23 ± 34.31	< 0.001

Data are presented as mean ± standard deviation

*P values represent comparisons between baseline and each postoperative time point using two-sided paired-samples t-tests

CST, central subfield thickness

**Table 3:** Best-corrected visual acuity (BCVA) at baseline and 12 months

Time point	BCVA (logMAR)	*P* value*
Baseline	1.08 ± 0.52	–
12 months	0.65 ± 0.42	<0.001

Data are presented as mean ± standard deviation

*P value represents the comparison between baseline and 12 months using the two-sided Wilcoxon signed-rank test

BCVA, best-corrected visual acuity; logMAR, logarithm of the minimum angle of resolution

### Longitudinal changes in OCT biomarkers

At baseline, EZ disruption, DRIL, and HRF were present in 18 (60.0%), 22 (73.3%), and 22 eyes (73.3%), respectively. At 12 months, the prevalence of DRIL decreased to 16 eyes (53.3%; *P* = 0.031), and HRF decreased to 10 eyes (33.3%; *P* < 0.001). EZ disruption decreased to 15 eyes (50.0%), although this change was not statistically significant (*P* = 0.250). Among eyes with the respective abnormalities at baseline, DRIL resolution occurred in 6 of 22 eyes (27.3%), HRF resolution in 12 of 22 eyes (54.5%), and restoration of EZ integrity in 3 of 18 eyes (16.7%). No new EZ disruption, DRIL, or HRF developed during follow-up.

### Unadjusted associations between baseline OCT biomarkers and outcomes

Eyes with intact baseline EZ showed numerically greater 12-month BCVA improvement than eyes with EZ disruption (0.63 ± 0.58 vs. 0.30 ± 0.30 logMAR), although the difference did not reach statistical significance (*P* = 0.093). Similarly, eyes without baseline DRIL showed greater numerical visual improvement than eyes with DRIL (0.78 ± 0.68 vs. 0.31 ± 0.27 logMAR; *P* = 0.077). Baseline HRF status was not associated with visual improvement (0.36 ± 0.11 vs. 0.46 ± 0.53 logMAR for eyes without and with HRF, respectively; *P* = 0.814) (Table 4). No significant differences in CST reduction were observed according to baseline EZ integrity or DRIL status. In contrast, eyes without baseline HRF showed a substantially greater unadjusted reduction in CST than eyes with HRF (398.00 ± 86.66 vs. 226.95 ± 81.73 μm; *P* < 0.001) (Table 5).

**Table 4:** Association of baseline OCT biomarkers with 12-month visual improvement

Baseline OCT biomarker	*n* (%)	BCVA improvement (logMAR)	*P* value*
EZ			
Intact Disrupted	12 (40.0) 18 (60.0)	0.63 ± 0.58 0.30 ± 0.30	0.093
DRIL			
Absent Present	8 (26.7) 22 (73.3)	0.78 ± 0.68 0.31 ± 0.27	0.077
HRF			
Absent Present	8 (26.7) 22 (73.3)	0.36 ± 0.11 0.46 ± 0.53	0.814

Data are presented as mean ± standard deviation or n (%)

BCVA improvement was defined as baseline minus 12-month logMAR BCVA

*P values were calculated using two-sided Mann–Whitney U tests

BCVA, best-corrected visual acuity; DRIL, disorganization of the retinal inner layers; EZ, ellipsoid zone; HRF, hyperreflective retinal foci; OCT, optical coherence tomography

**Table 5:** Association of baseline OCT biomarkers with 12-month CST reduction

Baseline OCT biomarker	*n* (%)	CST reduction, µm	*P* value*
EZ			
Intact	12 (40.0)	275.50 ± 122.95	0.912
Disrupted	18 (60.0)	270.61 ± 107.93	
DRIL			
Absent	8 (26.7)	226.13 ± 94.05	0.146
Present	22 (73.3)	289.45 ± 115.27	
HRF			
Absent	8 (26.7)	398.00 ± 86.66	< 0.001
Present	22 (73.3)	226.95 ± 81.73	

Data are presented as mean ± standard deviation or n (%)

CST reduction was defined as baseline minus 12-month CST

*P values were calculated using two-sided Welch’s t-tests

CST, central subfield thickness; DRIL, disorganization of the retinal inner layers; EZ, ellipsoid zone; HRF, hyperreflective retinal foci; OCT, optical coherence tomography

### Multivariable and sensitivity analyses

In multivariable analysis adjusted for baseline BCVA, concomitant phacoemulsification, and diabetic retinopathy stage, baseline DRIL was independently associated with worse 12-month BCVA (β = 0.399, 95% CI 0.035–0.762; *P* = 0.032). EZ disruption showed a similar direction of association but did not reach statistical significance after adjustment (β = 0.234, 95% CI − 0.045 to 0.514; *P* = 0.100). Baseline HRF was not independently associated with 12-month BCVA (β=−0.212, 95% CI − 0.524 to 0.100; *P* = 0.182) (Table 6).

**Table 6:** Multivariable analysis of baseline OCT biomarkers and 12-month functional and anatomical outcomes

Outcome	Baseline OCT biomarker	Adjusted β (95% CI)	*P* value
12-month BCVA, logMAR	DRIL present	0.399 (0.035 to 0.762)	0.032
12-month BCVA, logMAR	EZ disrupted	0.234 (− 0.045 to 0.514)	0.100
12-month BCVA, logMAR	HRF present	−0.212 (− 0.524 to 0.100)	0.182
12-month CST, µm	HRF present	15.3 (− 14.1 to 44.7)	0.309

BCVA models were adjusted for baseline BCVA, concomitant phacoemulsification, and diabetic retinopathy stage; the CST model was adjusted for baseline CST, concomitant phacoemulsification, and diabetic retinopathy stage

BCVA, best-corrected visual acuity; CI, confidence interval; CST, central subfield thickness; DRIL, disorganization of the retinal inner layers; EZ, ellipsoid zone; HRF, hyperreflective retinal foci; OCT, optical coherence tomography

Although the absence of baseline HRF was strongly associated with greater CST reduction in the unadjusted analysis, this association was attenuated after adjustment for baseline CST, concomitant phacoemulsification, and diabetic retinopathy stage. Baseline HRF was not independently associated with 12-month CST in the adjusted model (β = 15.3 μm, 95% CI − 14.1 to 44.7; *P* = 0.309) (Table 6).

In a sensitivity analysis additionally adjusting for baseline ERM stage, DRIL remained significantly associated with 12-month BCVA (β = 0.387, 95% CI 0.022–0.752; *P* = 0.038). Among the 18 eyes that were pseudophakic at baseline, the direction of the functional associations with EZ and DRIL was similar to that observed in the overall cohort, although neither reached statistical significance. BCVA improvement was 0.60 ± 0.54 versus 0.27 ± 0.27 logMAR in eyes with intact versus disrupted EZ (*P* = 0.263), and 0.74 ± 0.67 versus 0.29 ± 0.24 logMAR in eyes without versus with DRIL (*P* = 0.254). No association was observed between HRF status and BCVA improvement in this subgroup (*P* = 0.843). Concomitant phacoemulsification was not associated with greater visual improvement in the unadjusted comparison; mean BCVA improvement was 0.43 ± 0.52 logMAR in eyes undergoing combined phacoemulsification and 0.44 ± 0.43 logMAR in eyes undergoing PPV without combined phacoemulsification (*P* = 0.627).

### Postoperative treatment burden and safety

During postoperative follow-up, 22 eyes (73.3%) required no additional intravitreal treatment. A total of 19 postoperative intravitreal injections were administered, corresponding to a mean of 0.63 ± 1.43 injections per eye. No major intraoperative or postoperative complications, including retinal detachment, endophthalmitis, or clinically significant intraocular hemorrhage, were observed during follow-up.

## Discussion

In the present study, PPV combined with ERM and ILM peeling was associated with substantial anatomical and functional improvement in eyes with refractory DME. Macular thickness decreased markedly, visual acuity improved, and most eyes required no further intravitreal treatment during postoperative follow-up. The extent of visual recovery, however, varied according to the preoperative retinal microstructure. Among the OCT biomarkers evaluated, DRIL showed the most consistent relationship with postoperative visual outcome. Preserved EZ integrity was associated with a favorable, although less robust, trend toward visual improvement, whereas HRF did not provide independent prognostic information for anatomical outcome. These observations underscore the importance of retinal structural integrity in determining visual potential after surgery.

Our findings are consistent with previous studies demonstrating substantial anatomical improvement after vitrectomy for persistent or refractory DME, although the extent of visual recovery has varied considerably across reports [9, 20–23]. The surgical context differs across the literature, ranging from vitrectomy for nontractional DME to procedures incorporating membrane peeling, and these differences may contribute to the heterogeneity of reported functional outcomes. In our cohort, all eyes had advanced ERM and underwent both ERM and ILM peeling, representing a setting in which diabetic macular pathology coexisted with tangential mechanical distortion. Differences in the chronicity and severity of edema and in baseline retinal integrity may further influence the degree of postoperative visual recovery.

The effects of vitrectomy in refractory DME are likely multifactorial. Removal of the vitreous has been proposed to improve retinal oxygenation and modify the intraocular biochemical environment [10, 24]. ERM removal relieves tangential traction and mechanical distortion of the macula, while ILM peeling may further reduce residual tractional forces and remove a scaffold for cellular proliferation [25–31]. Together, these mechanisms may contribute to the sustained anatomical improvement observed after surgery.

Preoperative retinal microstructure may be particularly relevant to the degree of functional recovery. EZ integrity is an established structural correlate of photoreceptor function and has been associated with visual outcomes in DME across different treatment modalities [16, 32–34]. Eyes with preserved EZ in our cohort tended to achieve greater visual improvement than those with EZ disruption, although the association was not sufficiently robust to establish EZ integrity as an independent prognostic marker. Nevertheless, the direction of this relationship is biologically plausible, as preservation of the outer retinal architecture may reflect greater functional reserve. Conversely, established photoreceptor disruption may constrain visual recovery even when macular thickness improves substantially. DRIL showed the strongest and most consistent relationship with postoperative visual outcome. This OCT feature reflects disruption of the normal organization of the inner retinal layers and has been closely associated with visual dysfunction in DME [13, 16, 35]. Eyes with baseline DRIL had poorer visual outcomes, supporting the concept that inner retinal integrity is an important determinant of functional recovery after surgery. In eyes with ERM, tangential traction may itself contribute to inner retinal distortion; therefore, DRIL in this setting may represent the combined structural consequences of diabetic neuroretinal injury and mechanical deformation. Its consistent association with postoperative vision nevertheless indicates that DRIL captures clinically meaningful retinal damage relevant to visual prognosis.

The postoperative evolution of the OCT biomarkers was not uniform. DRIL became less frequent during follow-up, whereas restoration of EZ integrity occurred in relatively few eyes. This pattern may indicate that some changes in inner retinal organization associated with chronic edema or mechanical distortion are more reversible than established photoreceptor damage. Such differences in structural recovery may partly explain why marked reduction in macular thickness does not invariably translate into a comparable degree of visual improvement.

HRF showed a different pattern. HRF have been proposed as imaging markers of inflammatory activity in DME and have been associated with disease severity and treatment response [15, 36]. Although eyes without baseline HRF showed greater CST reduction in the unadjusted analysis, this association was attenuated after adjustment for baseline CST and relevant clinical factors. HRF therefore did not emerge as an independent marker of postoperative anatomical recovery. This observation highlights the importance of considering baseline macular morphology when interpreting the magnitude of retinal thickness reduction.

Another clinically relevant finding was the low requirement for additional intravitreal treatment during postoperative follow-up. Nearly three-quarters of eyes required no further intravitreal therapy. For patients with refractory DME who have previously undergone repeated injections, such a postoperative course may be clinically meaningful. However, because retreatment decisions were individualized and the study lacked a medically treated comparison group, these observations should not be interpreted as evidence of a treatment-sparing effect attributable to surgery.

The strengths of this study include a well-defined cohort of eyes with refractory DME and advanced ERM, a standardized surgical technique, uniform OCT imaging, and systematic assessment of retinal microstructural biomarkers. Several limitations should also be considered. The retrospective, single-center design and modest sample size limit statistical power and generalizability. The absence of a nonsurgical control group precludes causal attribution of the observed anatomical and functional changes to surgery. The sample size also restricted the complexity of multivariable modeling, and residual confounding or overlap among OCT biomarkers cannot be excluded. In addition, advanced ERM may itself alter inner retinal morphology, which should be considered when interpreting structural biomarkers such as DRIL in this population.

In conclusion, PPV with ERM and ILM peeling was associated with substantial anatomical and functional improvement in eyes with refractory DME and advanced ERM. The degree of visual recovery appeared to depend not only on resolution of macular edema but also on the integrity of the underlying retinal architecture. Among the OCT biomarkers evaluated, DRIL provided the most consistent prognostic information for postoperative visual outcome, whereas the associations of EZ integrity and HRF were less robust. Assessment of preoperative retinal microstructure, particularly DRIL, may therefore complement conventional clinical and OCT parameters when estimating visual prognosis and counseling patients considered for surgery.

## Data Availability

The datasets generated and/or analyzed during the current study are not publicly available due to patient privacy and institutional data protection requirements but are available from the corresponding author on reasonable request.
